# TRIM38 triggers the uniquitination and degradation of glucose transporter type 1 (GLUT1) to restrict tumor progression in bladder cancer

**DOI:** 10.1186/s12967-021-03173-x

**Published:** 2021-12-14

**Authors:** Xiaojing Wang, Hongchao He, Wenbin Rui, Ning Zhang, Yu Zhu, Xin Xie

**Affiliations:** Department of Urology, Ruijin Hospital, School of Medicine, Shanghai Jiaotong University, 197 Rui Jin 2nd Road, Shanghai, China

**Keywords:** TRIM38, GLUT1, Glycolysis, BAY-876, Bladder cancer

## Abstract

**Background:**

Loss-of-function mutations or abnormal expressions of E ubiquitin ligases contributes to tumorigenesis. TRIM38 was reported to regulate immunity, inflammatory responses or apoptosis, but its roles in tumor progression remain inconclusive. This study aimed to investigate the functional roles of TRIM38 in bladder cancer to identify effective targets.

**Methods:**

Firstly, the expression data of ubiquitination-associated genes were derived from the TCGA-BLCA cohort. Univariate Cox regression method was utilized to screen prognostic genes. Colony formation assay, Transwell assay, sphere formation assays were used to assess functional roles of TRIM38. TAP/MS assay was used to identify downstream substrates of TRIM38. Fresh clinical BLCA tissues were collected to evaluate the clinicopathological features of patients with different TRIM38 expression. The subcutaneous tumor models were established to determine the drug efficacy of BAY-876.

**Results:**

A list of ubiquitination-associated signature was identified based on the screening in TCGA-BLCA cohort. Subsequent validations revealed that TRIM38 was a significant suppressor in tumors, which was expressed lowly in BLCA. Kaplan–Meier analysis and correlation analysis suggested that patients with low TRIM38 expressions had shorter survival time and advanced clinical characteristics. Targeting TRIM38 reinforced BLCA cells proliferation, migration and stemness. Mechanistically, TRIM38 interacted with GLUT1, thereby promoting its ubiquitinoylation and degradation. Furthermore, TRIM38 deficiency relied on accumulated GLUT1 proteins to enhance BLCA malignant features and cellular glycolytic capacity. We accordingly investigated the efficacy of GLUT1 inhibitor (BAY-876) in BLCA and determined its IC50 values across cell lines. Tumor xenograft models further validated that BAY-876 could effectively suppress the in vivo growth of TRIM38^low/−^ BLCA.

**Conclusions:**

Our results suggested that TRIM38 plays a tumor suppressive role in BLCA pathogenesis and TRIM38/GLUT1 axis is a therapeutic vulnerability for clinical treatment, which possessing great translational significance.

**Supplementary Information:**

The online version contains supplementary material available at 10.1186/s12967-021-03173-x.

## Background

In the urological system, bladder urothelial carcinoma (BLCA) is one of the most common and lethal malignancies worldwide with a relatively high incidence and mortality rate [1–3]. According to the newest statistics in 2021, the estimated new BLCA cases originating from the urinary bladder were 83,730, and the estimated deaths were 17,200 in the United States [4]. According to the extent of the tumor cells invading into the muscle layer, BCLA could divided into nonmuscle-invasive bladder cancer (NMIBC), accounting for nearly 75% of patients, and the remaining cases of muscleinvasive bladder cancer (MIBC) [5, 6]. Nevertheless, NMIBC patients have a high risk of developing into stages with invasive bladder tumor [7, 8]. MIBC is a life-threatening disease with only 6% of 5-year survival rate, which leads to almost 100% of death from this disease [9, 10]. Currently, the main strategies for advanced BLCA are ineffective, and the clinical grades or stages could hardly predict the early metastasis [11, 12]. The treatment technologies were reported to be improved continuously, including surgery, chemotherapy, and immunotherapy [13, 14]. However, the overall survival rate remains still low owing to genetic heterogeneity and various clinical characteristics [15]. As a result, an in-depth study of the potential molecular mechanisms involved in BLCA is urgently warranted to identify more effective predictors and more efficient anticancer treatments.

Aberrant epigenetic modifications represent a molecular feature of multiple malignancies, including promoter methylation, histone acetylation, as well as ubiquitination of proteins [16, 17]. The ubiquitin–proteasome system (UPS) plays the main roles of modulating proteins turnover, which is tightly associated with metabolic disorders, cardiovascular diseases, neurological disorders and tumorigenesis [18, 19]. Although previous studies have already explored the abnormal ubiquitination mechanisms of identified onco-proteins within the progression of BLCA, the associations between UPS and BLCA were still indefinite [20]. Tripartite motif protein 38 (TRIM38), an E3 ubiquitin ligase, belongs to the TRIM protein family that participates in multiple biological processes, including cell apoptosis, innate immunity and inflammation processes [21, 22]. This gene encodes protein contains a RING-type zinc finger, B box-type zinc finger and SPRY domain. Previous documents have found that TRIM38 could mediate the degradation of TAB2/3, two cellular components required for TNFα- and IL-1β-triggered cellular response, to negatively regulate inflammatory response [22]. Besides, TRIM38 could catalyze K48-linked polyubiquitination of the TLR3/4 adapter protein TIR domain-containing adapter-inducing IFN-β, which triggering its proteasomal degradation in immune cells [23]. However, the underlying mechanisms between aberrant TRIM38 and cancer progression were never been reported. Given that we have found that TRIM38 exhibits low expression levels in BLCA, the potential reasons were indefinite.

Tumor progression depends on the reprogramming of cellular metabolism as both direct and indirect consequence of oncogenic mutations [24, 25]. The key characteristic of cancer cell metabolism is the efficiency to obtain essential nutrients from microenvironment and adopt the nutrients to maintain cell viability and create new biomass. Aerobic glycolysis, also named as the Warburg effect, is considered to be a prominent hallmark in tumor cells, which is depicted by elevated glucose uptake and lactate production in the normal oxygen conditions [26]. As well documented, the high glycolytic flux in tumor cells relys on glycolysis-associated signature, including GLUT3, SRC-3, hexokinase 1 (HK1), as well as lactic dehydrogenase A (LDHA), leading to production of pyruvate, alanine, and lactate [27, 28]. These functions is primarily controlled by the glucose transporters family (GLUT1-14). Among these GLUT members, GLUT1 is the most extensively explored in various malignancies, like prostate cancer, renal cell carcinoma, gastric cancer, as well as bladder cancer [29, 30]. Chen et al. [31] have observed that SIRT1 may interact with GLUT1 to modulate the proliferation and glycolysis phenotype in bladder cancer. However, the reasons that account for aberrantly high GLUT1 protein levels in BLCA were still indistinct to explain.

In the study, our team identified for the first time that TRIM38 is a tumor suppressor in BLCA pathogenesis and demonstrated the biological roles of TRIM38 in BLCA through in vitro and in vivo experiments. We confirmed that GLUT1 was the downstate substrate of TRIM38 to drive BLCA progression. Overall, our study highlighted that TRIM38/GLUT1 axis is a potential therapeutic vulnerability for BLCA.

## Methods and materials

### Cell lines and culture

The human BLCA cell lines (T24, EJ and 5637) and 293 T cells were obtained from the American Type Culture Collection (ATCC). The 293 T and EJ cells were maintained in DMEM (Invitrogen) supplemented with 10% fetal bovine serum (H Clone) and 1% antibiotics. Besides, the T24 and 5637 cells were cultured in the RPMI 1640 (Invitrogen, Carlsbad, CA, USA) added with 10% fetal bovine serum (HyClone). All cells were maintained in a humidified incubator at 37 °C under the 5% CO_2_.

### Collection of BLCA patient samples

BLCA samples were obtained via surgical resection from patients between February 2018 and July 2020 at the department of urology, Ruijin hospitcal (Shanghai, China). None of these patients suffered from distal metastasis, and postoperative pathology validated that the paraffin samples were all urothelial carcinoma. Other cases with carcinoma in situ were all excluded via the confirmation by two independent pathologists. All patients have signed the informed consent forms before the research. The use of human BLCA tissues in the study were approved by the medical ethics committee of Ruijin hospital. Lastly, we also downloaded the expression data and corresponding clinical information of BLCA patients from the public datasets, including TCGA-BLCA cohort (https://portal.gdc.cancer.gov/), GSE13507 dataset (https://www.ncbi.nlm.nih.gov/gds/?term=GSE13507) and GSE (https://www.ncbi.nlm.nih.gov/gds/?term=GSE32548). The clinical characteristics of samples contained age, gender, tumor grades and AJCC stages.

### Generation of stable *CRISPR/Cas9*-mediated TRIM38-knockout cells

We utilized the pX459 plasmid to clone guide oligos to target TRIM38 gene in BLCA cell lines. T24 and EJ cells were plated into the culture dish and then transfected with the pX459 plasmids for 24 h. Subsequently, 1 μg/mL puromycin was utilized to screen cells for 3 days. Afterwards, the left living cells were seeded in 96 well plate via limited dilution to have the monoclonal cell line. The TRIM38 knockout cell clones are lastly confirmed by western blot and validated by sanger sequencing. The specific sequences that target TRIM38 were listed as the following: sgTRIM28#1:F: 5′-CACCGGCCCCTCGTCTTCGCAGAAC-3′; sgTRIM28#1:R: 5-AAACGTTCTGCGAAGACGAGGGGCC-3′. sgTRIM28#2:F: 5-CACCGAACAGACTCTGAGTAGACTG-3′; sgTRIM28#2:R: 5-AAACCAGTCTACTCAGAGTCTGTTC-3′.

### TRIM38 overexpression and knockdown

Lentiviral vectors containing TRIM38 shRNA were purchased from Genchem (Shanghai, China). Cells were seeded into a 6-cm dish with a density of 3 × 10^6^ cells. After 12 h, cells were transfected with the overexpression plasmids (2 μg) or shRNA (1.5 μg) targeting specific gene using Lipofectamine® 2000 reagent (Invitrogen) following the manufacturer’s instructions.

### CCK-8, cell proliferation assay and 3D soft agar assay

The MTT assay was selected to evaluate the viability of bladder cancer cells. Cells were cultured in 96-well plates with density of 5 × 10^4^ cells/well. Then, the 10 μL MTT was added to the fresh medium to replace the old medium after 24 h when the cells were attached. The medium was changed after incubation with the medium containing MTT at 37 °C for 4 h, and the DMSO was added. Lastly, the absorbance was measured at 490 nm. The results are determined as percentage inhibition compared to the control group. The Cell Counting Kit-8 (CCK-8) kit (Dojindo) was utilized to detect the cell proliferation rate according to the manufacturer’s protocol. First of all, cells were plated into the 96-well dishes with a denstity of about 1000 cells per well. Then, 10 μL of the CCK-8 solution was supplemented into the cell culture, and incubated for 2 h during a period of day 2 to day 5. Lastly, the resulting color was detected at OD 450 nm with a microplate absorbance reader (Bio-Rad). Each assay was carried out in triplicate. For the colony formation assay, EJ and T24 cells with WT TRIM38 or TRIM28 deficiency were plated into the 6-wells plates with a density of 600 cells per well. After 2 weeks, the formed cell colonies were fixed with 4% polyformaldehyde for 30 min and stained with 0.1% crystal violet (Servicebio) for another 20 min at room temperature. Each clone formation assay was carried out in triplicate. For the soft agar assay, 1.4% noble agar was firstly melted and then cooled to 40 °C by a water bath. Then, the base layer was added to each well for anchorage independent growth. The plates were remained at the room temperature for 20 min to let the agar to solidify. Lastly, 1 mL of 0.35% agar/medium with 4000 cells was put on the top layer of agar. After 21 days, the colonies were counted with a light microscope. Each assay was carried out in triplicate.

### Transwell assays

Briefly, 5 × 10^4^ TRIM38-WT or TRIM38-KO cells (EJ, T24) were seeded into the inside of the chamber with Matrigel-coated Boyden invasion chambers (BD Biosciences) within 200 μL of serum-free RPMI 1640 medium. Afterwards, 800 µL of 1640 medium mixed with 10% FBS were supplemented into the outside of the chamber, which was placed in a 24-well plate. After incubation for 24 h under the 37 °C condition, the outside membrane was transferred into a new 24-well plate with 4% paraformaldehyde and stained with the crystal violet (Sigma). Each assay was carried out in triplicate.

### Real-time reverse transcription PCR (qRT-PCR)

Total RNA was isolated from bladder cancer cells using the TRIzol reagent (Tiangen), and cDNA was reversed-transcribed using the Superscript RT kit (TOYOBO) following the manufacturer's instructions. An equal amount of RNA (2 μg) was reverse transcribed into complementary DNA (cDNA) using Superscript Reverse Transcriptase (Applied Biosystems, USA). Quantitative real-time PCR (qRT-PCR) was performed on the ABI 7500 real-time PCR system (Applied Biosystems, USA). All quantitations were normalized to the level of endogenous control GAPDH.

### In vivo ubiquitination assay

293T cells were transfected with HA-ubiquitin and the indicated constructs. 36 h after transfection, cells were treated with 30 μM MG132 for 6 h and then lysed in 1% SDS buffer (Tris [pH 7.5], 0.5 mM EDTA, 1 mM DTT) and boiled for 10 min. For immunoprecipitation, the cell lysates were diluted tenfold in Tris–HCl buffer and incubated with anti-GLUT1 antibody for 4 h at 4 °C. The bound beads are then washed four times with BC100 buffer (20 mM Tris–Cl, pH 7.9, 100 mM NaCl, 0.2 mM EDTA, 20% glycerol) containing 0.2% Triton X-100. The ubiquitinated form of GLUT1 was detected by Western blot using anti-HA antibody.

### Immunohistochemistry (IHC)

Immunostaining assay was conducted on resected BLCA samples from selected patients with the specific antibodies for TRIM38 (AF0307) and GLUT1 (ab115730), respectively. Briefly, the BLCA tissues were hydrated with gradient alcohols and then water. The antigen retrieval solution was carried out in a microwave oven for 30 min. Afterwards, 3% H_2_O_2_ was used to block endogenous peroxidase on the slides. Then, the slides were incubated with specific antibodies overnight. After washing for three times, the slides were incubated with poly-HRP-conjugated anti-Rabbit IgG (SA1022, BOSTER). The IHC images were captured with an Olympus FSX100 microscope (Olympus, Japan).

### Animal experiments

All animal experiments were approved by the Animal Care Committee of Shanghai Ruijin hospital. Four-week-old male nude mice from the Institute of Zoology (Beijing, China) were selected to establish the tumor xenografts and randomly divided into two groups (N = 5 per group). TRIM38-KO EJ cells or Ctrl cells were subcutaneously injected into the right flanks of the nude mice (2 × 10^6^, 200 µL), respectively. The tumor volumes and weight were detected at 1–4 weeks after injection and calculated as the length × width^2^ × 0.5.

### Statistical analysis

The Student’s t test was used to evaluate differences between two groups, while the log-rank test was utilized to analyze the differences in survival. All experimental data were indicated as mean ± standard deviation (SD). Statistical analysis was carried out using the GraphPad Prism 7.0 software. A value of *P* < 0.05 was regarded to be statistically significant.

## Results

### Identification of E3 ubiquitin ligase TRIM38 as a pivotal suppressor in BLCA

**Fig. 1:**
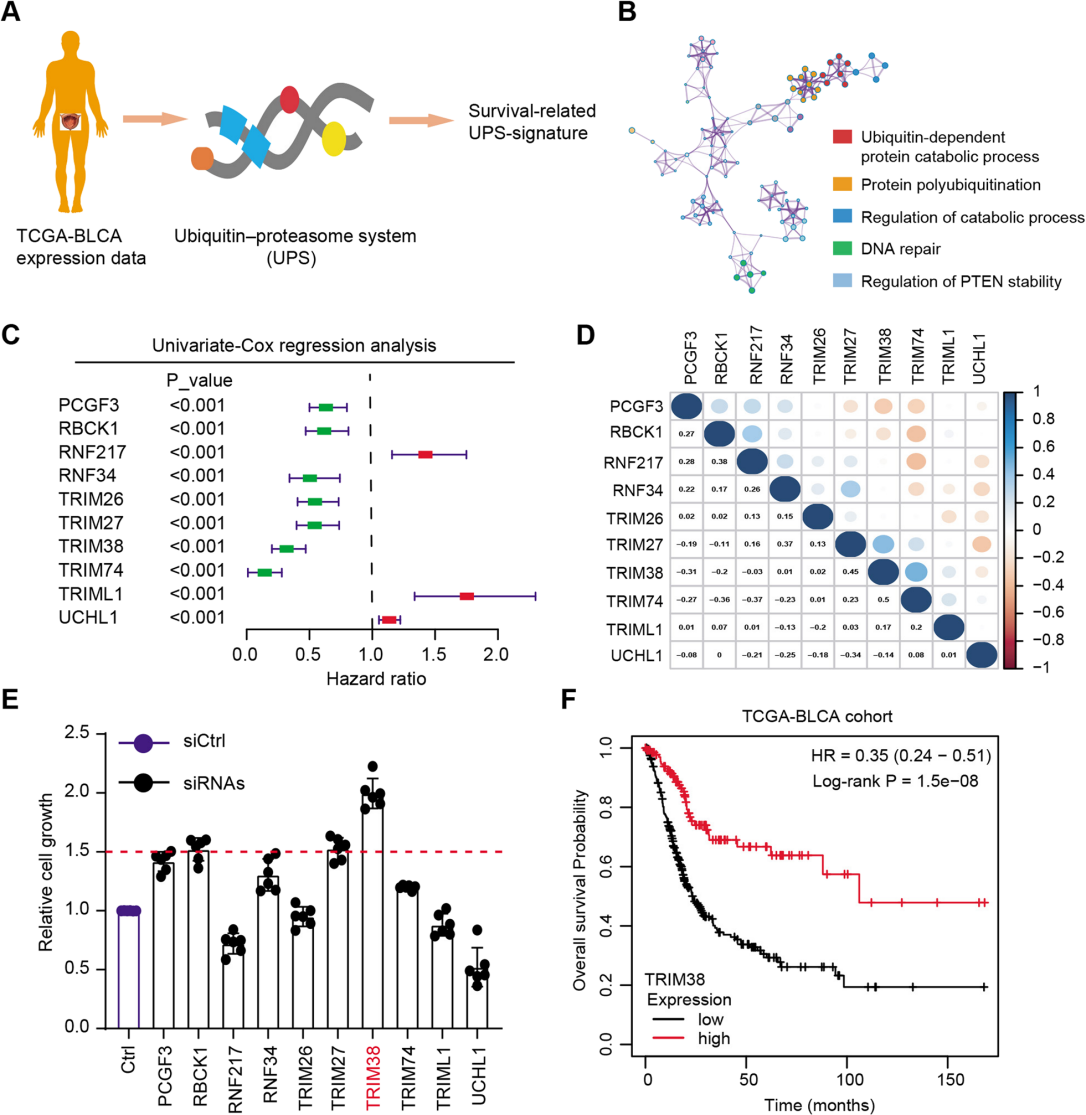
High-throughput sequencing screens and experimental validations highlighted that TRIM38 was a tumor suppressor in BLCA. **a** Schematic diagram showing the screening process of prognostic UPS-signature in BLCA. **b** Gene ontology (GO) analysis revealed the enriched crosstalk based on the prognostic UPS-signature. **c** Forest plot illustrating the hazard and favorable targets in BLCA, where red genes represented the risk factors and green genes represented the protective factors. **d** Correlation heatmap showing the selected 10 genes in BLCA, where blueness exhibited the positive associations and brownness showed the negative relationships. **e** MTT assays validated the functional roles of indicated 10 genes via transfection with individual siRNAs, respectively. **f** Kaplan–Meier analysis indicated that BLCA patients with low TRIM38 levels had a higher risk of tumor progression relative to those with high TRIM38 expressions, as indicated by the log-rank test

We collected a list of 478 ubiquitin–proteasome system (UPS)-related genes and extracted the corresponding expression data from the TCGA-BLCA cohort (Fig. 1A and Additional file 1: Table S1). Univariate Cox regression analysis was conducted to screen a series of prognosis-associated UPS genes with *P* < 0.05 (Additional file 2: Table S2). Gene ontology (GO) analysis was then performed based on these genes and several biological enriched items were highlighted, including ubiquitin-dependent protein catabolic process, regulation of catabolic process or DNA repair crosstalk (Fig. 1B). We further illustrated the top 10 prognosis-associated UPS-signature via forest plot, where the hazard factors were noted in red and favorable factors were noted in green (Fig. 1C). We observed that high levels of RNF217, TRIML1 or UCHL1 correlated with worse outcomes, whereas TRIM38, RBCK1 or TRIM26 were favorable genes in BLCA (Fig. 1C). The underlying associations across the 10 genes were illustrated through the correlation heatmap (Fig. 1D). As shown by the MTT assay, TRIM38 was the most potent hit and targeting TRIM38 resulted in the most increase of cell growth compared with other candidates (Fig. 1E). Lastly, we divided the BLCA samples into TRIM38^high^ and TRIM38^low^ groups according to the median data and found that patients with low TRIM38 levels had the worse overall survival (OS) outcomes relative to those with high TRIM38 levels (Fig. 1F). Collectively, these findings indicated that TRIM38 might be a tumor suppressor in BLCA that deserves to be further investigated.

### TRIM38 expressed lowly in BLCA which predicts an unfavorable prognosis

**Fig. 2:**
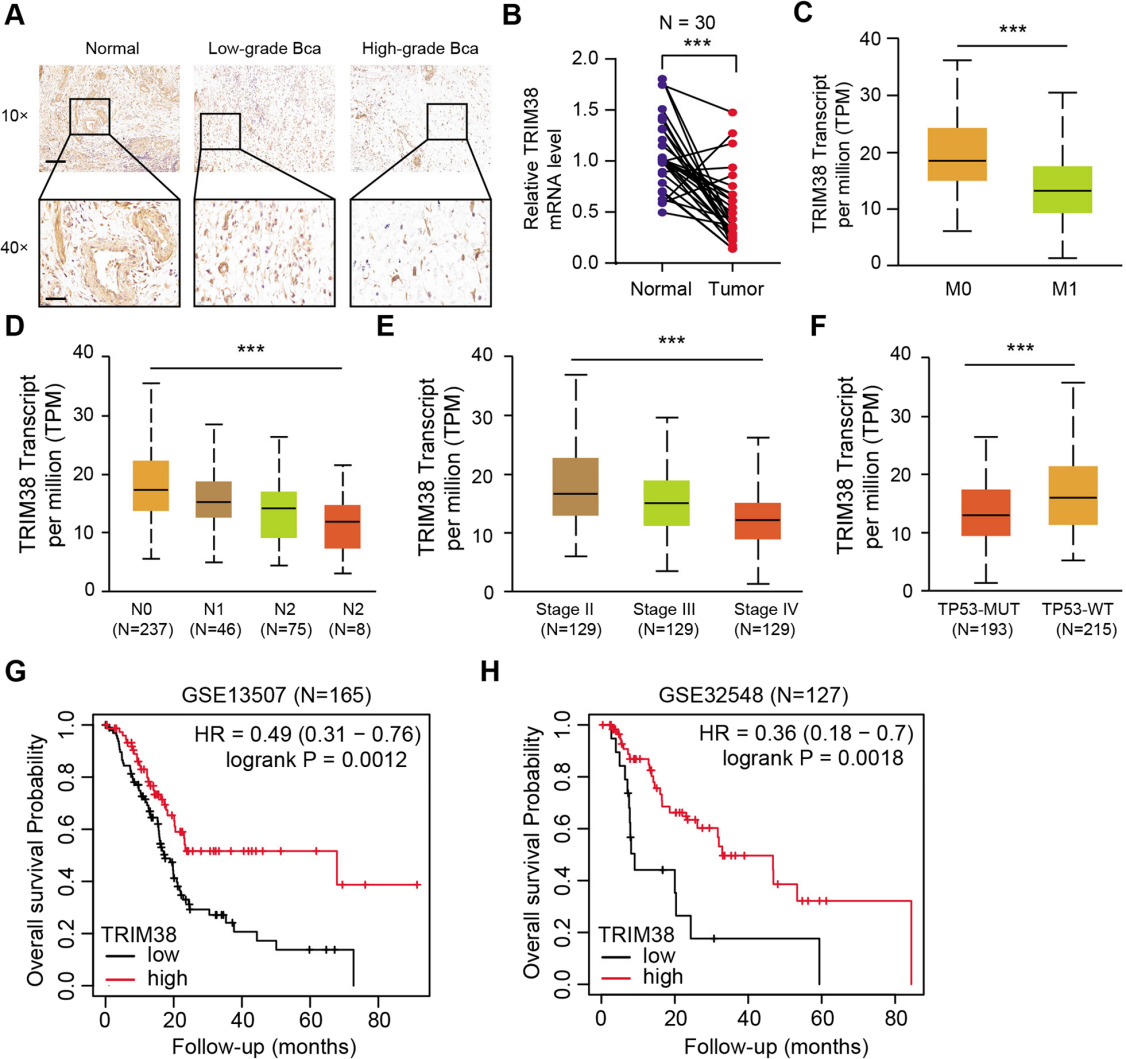
TRIM38 expressed lowly in BLCA and down-regulated TRIM38 correlated with advanced clinical characteristics and poor prognosis. **a** Representative immunohistochemistry (IHC) graphs showing the staining levels of TRIM38 in patients with different tumor grades. scale bars = 100 μm (upper), scale bars = 20 μm (lower). **b** The results from qRT-PCR analysis exhibited that the TRIM38 mRNA levels were notably upregulated in BLCA samples relative to their adjacent normal tissues. β-actin was detected as the loading control. **c**–**f** Low TRIM38 expression levels correlated with tumor metastatic stages, lymph node metastasis, clinical pathological stages and TP53 mutation status. **g**, **h** Other BLCA datasets (GSE13507 and GSE32548) were utilized to suggest that patients with low TRIM38 expressions suffered from shorter overall survival (OS) probability compared with patients with high TRIM38

To further confirm the clinical significance of TRIM38 in BLCA, we collected totally 30 tumor samples with matched normal tissues from the department of urology, Shanghai Ruijin Hospital. We found that TRIM38 protein levels were significantly lower in tumor samples than normal tissues via immunohistochemistry (IHC) (Fig. 2A). The mRNA levels of TRIM38 were also significantly down-regulated in tumor samples (Fig. 2B). Moreover, low TRIM38 levels correlated with advanced TNM stages, metastasis, pathological stages and TP53 mutation (Fig. 2C–F). Lastly, we conducted the Kaplan–Meier analysis in another two BLCA cohorts and found that low TRIM38 levels correlated with worse OS outcomes in GSE13507 (N = 165, log-rank test *P* = 0.0012) and GSE32548 (N = 127, log-rank test *P* = 0.0018) (Additional file 3: Table S3). Taken together, we confirmed that low TRIM38 could predict worse survival outcomes in BLCA samples, which was an independently hazard biomarker.

### TRIM38 suppresses cell proliferation, migration and stemness features of BLCA

**Fig. 3:**
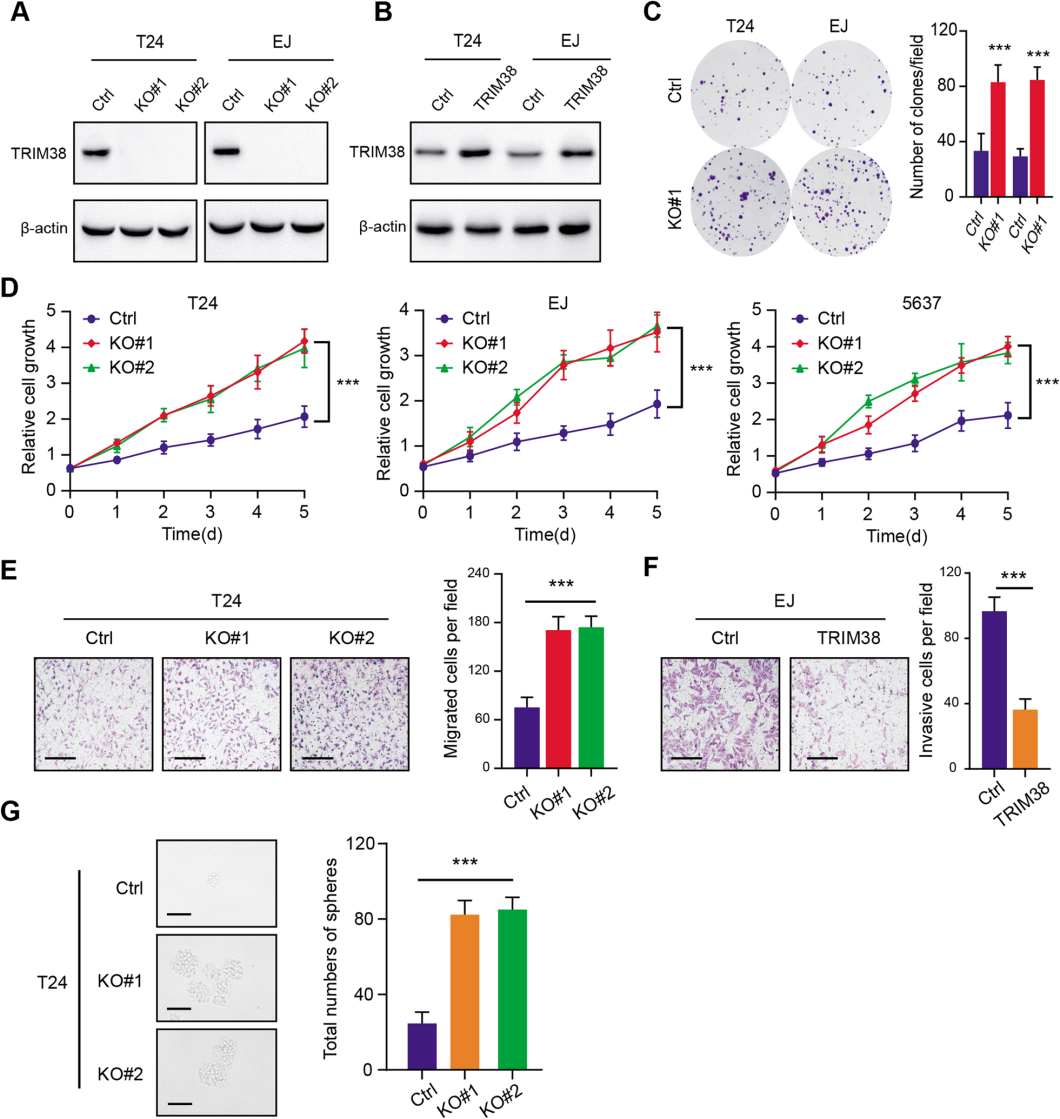
TRIM38 inhibition enhanced BLCA proliferation, migration and stemness features. **a** Western blot assay exhibiting the knockout efficiency of TRIM38 in T24 and EJ cells. **b** Western blot assay showing the overexpression of TRIM38 in T24 and EJ cells. **c** Colony formation capacity was determined in Ctrl and TRIM38-KO cells (T24 and EJ). Quantification of results were showed on the right panel. **d** CCK-8 assays were conducted to exhibit the cell growth in the indicated cells (T24, EJ, 5637) at every timepoints. **e** Transwell assays showed that the invasive capacity of BLCA cells was enhanced with TRIM38 ablation (left panel). Quantification of results was exhibited on the right panel. Scale bars = 250 μm. **f** The invasive ability of EJ cells was suppressed when cells were transfected with TRIM38 (left panel). Quantification of results was exhibited on the right panel. Scale bars = 250 μm. **g** Sphere formation assay was conducted to confirm that TRIM38 deficiency could notably drive stemness features of T24 cells. Quantification of results was exhibited on the right panel. Scale bars = 200 μm

To examine the functional roles of TRIM38 in BLCA, we established the stable TRIM38-overexpressing BLCA cells (T24 and EJ) and TRIM38 knockout cells via *CRISPR/Cas9* technology. The corresponding protein levels were confirmed by western-blot (Fig. 3A). Besides, stable BCLA cells with TRIM38 overexpression were also established via lentivirus infection, which were detected by western blot (Fig. 3B). TRIM38 deficiency notably increased the colony formation efficiency of T24 and EJ, as indicated by the clone numbers (Fig. 3C). Meanwhile, knockout of TRIM38 significantly enhanced the cell proliferation rates in T24, EJ and 5637 cells, as implicated by the CCK-8 assays (Fig. 3D). In addition, TRIM38 loss also promoted the migration and invasion abilities of T24 and EJ cells, whereas TRIM38 overexpression suppressed the migration capacity of cells (Fig. 3E, F). The self-renewal ability of cells was also remarkably increased in TRIM38-deficienct cells compared with controls, as indicated by the sphere numbers and sizes (Fig. 3G). Collectively, our results indicated that TRIM38 deficiency could remarkably promote BLCA progression, including cell growth, migration and stemness features.

### TRIM38 recognizes and triggers ubiquitin-dependent degradation of glucose transporter type 1 (GLUT1)

**Fig. 4:**
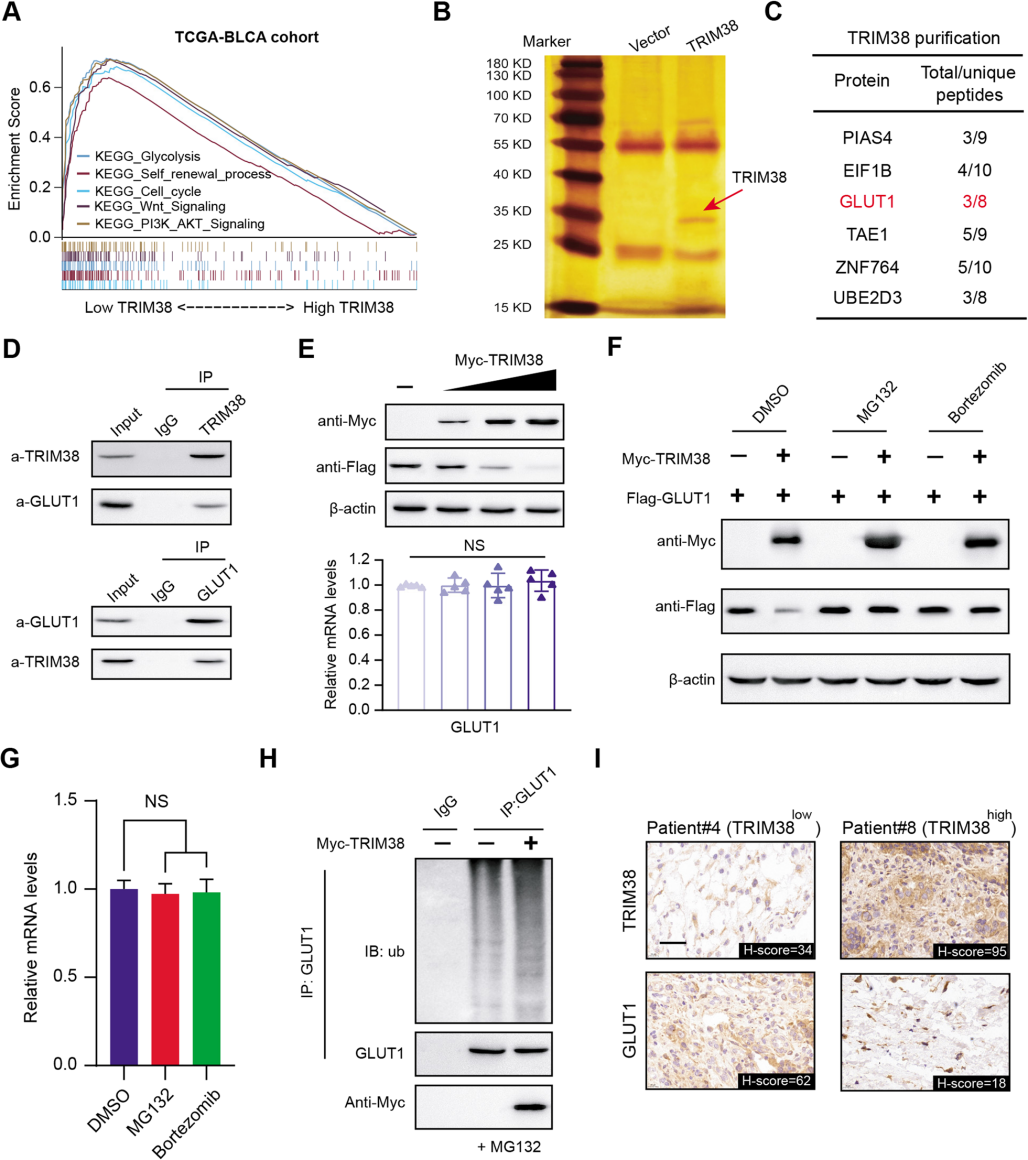
TRIM38 recognizes glucose transporter type 1 (GLUT1) and triggers ubiquitin-dependent degradation. **a** Gene Set Enrichment Analysis (GSEA) exhibited the enriched crosstalk in TRIM38-high versus TIM38-low samples. **b** Purification of TRIM38 proteins was conducted to identify the potential interacting substrates. The potential candidates with high frequency of enriched peptides were listed on the right table (**c**). **d** Western blot of indicated proteins in WCLs and Co-IP samples of IgG or anti-TRIM38 antibody obtained from the cell extracts of T24 cells treated with 20 μM of MG132 for 8 h. **e** Western blots of indicated proteins in WCLs from EJ cells transfected with an increasing amount of Myc-TRIM38 plasmids. The quantification of relative mRNA levels of GLUT1 was shown below, where the cells in the control group were transfected with the vector plasmid. **f** Western blot of GLUT1 and TRIM38 proteins in WCLs from T24 cells transfected with indicated plasmids with DMSO, MG132 (20 μM) or with Bortezmib (20 nM) for 12 h. **g** RT-qPCR assessment of GLUT1 mRNA expression in T24 cells treated with DMSO, MG132 (20 μM) or with Bortezmib (20 nM) for 12 h. The mRNA level of GAPDH was used for normalization. Data are shown as means ± SD (N = 3). The cells in control groups were treated with DMSO. **h** Western blot of the proteins of ubiquitination assays from T24 cells transfected with the indicated plasmids and treated with 20 μM MG132 for 12 h. **i** Representative IHC results exhibiting the negative associations between TRIM38 and GLUT1 proteins. Scale bars = 20 μm

To further investigate the potential mechanisms of tumor progression mediated by TRIM38 deficiency, we conducted the Gene Set Enrichment Analysis (GSEA) in TCGA-BLCA dataset based on the expression data matrix. We found that several significant biological items were notably enriched in TRIM38-low samples, including glycolytic process, cell cycle, Wnt signaling and PI3K-AKT signaling (Fig. 4A). We then overexpressed TRIM38 tagged with Flag in T24 cells. Tandem Affinity Purification (TAP) method was utilized to purificate the TRIM38-containing protein complex and we detected the proteins in the complex via mass spectrometry (MS) technology (Fig. 4B). Given the tight associations between TRIM38 and glycolysis, we detected several glycolytic associated genes in the complex, and GLUT1 was the top hit (Fig. 4C). We then performed Co-IP using the anti-TRIM38 antibody and anti-GLUT1 antibody in T24 cell lysate, individually. TRIM38 was able to immunoprecipiate GLUT1 (Fig. 4D). Reciprocally, GLUT1 was able to immunoprecipiate TRIM38, indicating the endogenous interactions between them (Fig. 4D). Besides, we co-transfected the T24 cells with Myc-TRIM38 and Flag-GLUT1 and observed that GLUT1 protein levels, not the mRNA levels, significantly decreased in a dose-dependent manner with an increase amount of TRIM38 (Fig. 4E). Considering that TRIM38 is an E3 ubiquitin ligase that mediates degradation of substrates, we observed that TRIM38 could markedly decrease GLUT1 protein levels, and this effect was completely reversed by treatment with proteasome inhibitors MG132 or Bortezomib (Fig. 4F). Moreover, we did not find significant associations between TRIM38 and GLUT1 mRNA levels in the TCGA-BLCA cohort (Additional file 4: Fig. S1A, B). In line with the above results, no alterations of GLUT1 mRNA levels were detected across the indicated groups, indicating that TRIM38 may regulate GLUT1 stability via posttranscriptional modifications (Fig. 4G). Furthermore, expression of wild-type (WT) TRIM38 could indeed induce the robust polyubiquitination of GLUT1 via in vivo ubiquitination assays (Fig. 4H). Lastly, we also performed IHC analysis in the BLCA tumors in Ruijin hospital and confirmed the negative associations between the two proteins in 40 samples (Fig. 4I). In conclusion, we demonstrated that TRIM38 E3 ubiquitin ligase regulates GLUT1 protein stability through ubiquitin-dependent proteasomal degradation in bladder cancer cells.

### TRIM38 depended on GLUT1 to modulate glycolytic process and tumor progression

**Fig. 5:**
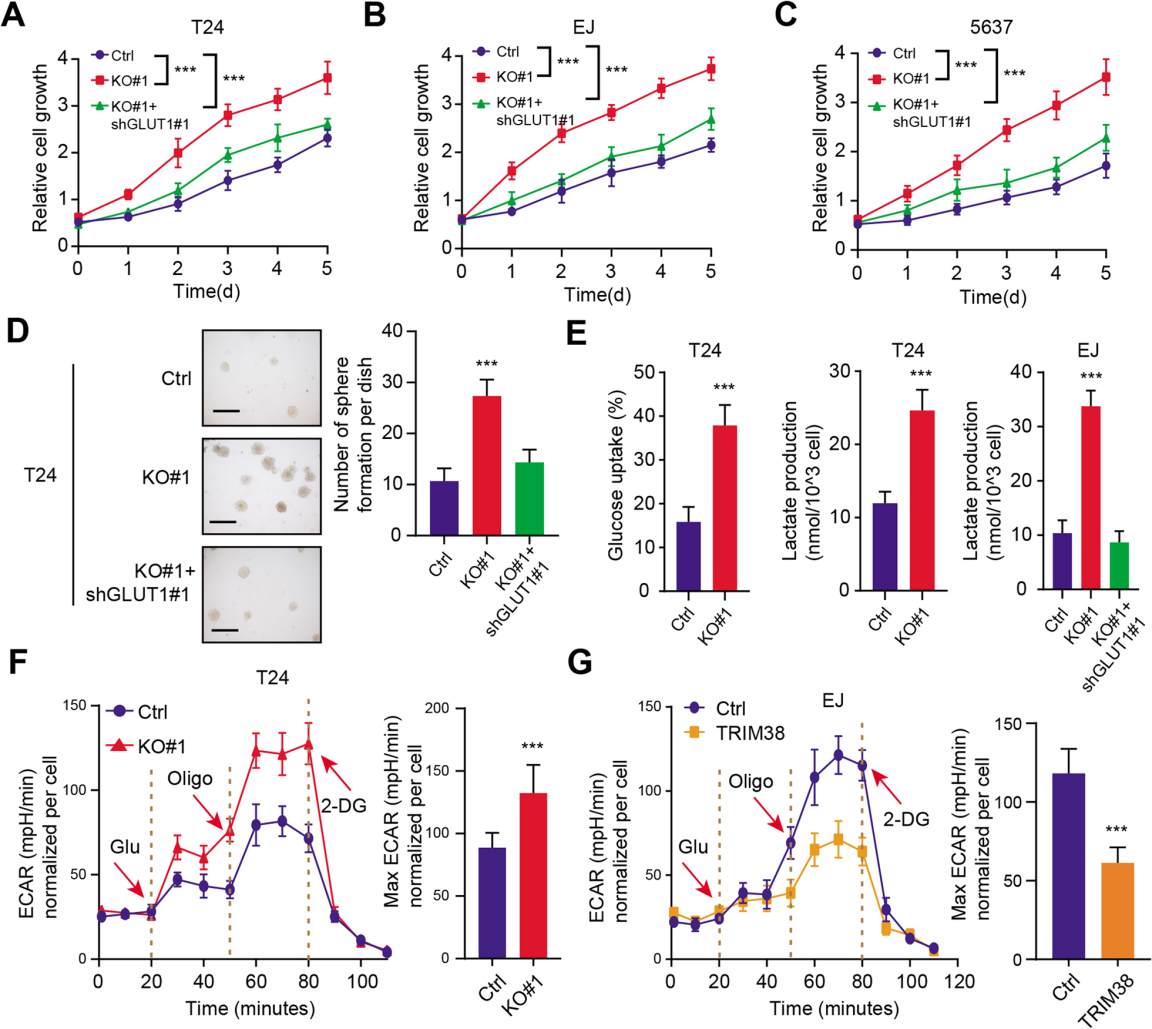
Down-regulated TRIM38 depended on GLUT1 to drive glycolytic process and tumor progression. **a**–**c** Cell viability was evaluated by Cell Counting Kit-8 (CCK-8) assay, in which GLUT1 knockdown suppressed the growth of TRIM38-deficient cells. **d** TRIM38 knockout enhanced T24 cells anchorage-independent growth in soft agar, which could be notably suppressed by TRIM38 overexpression (scale bars = 200 µm, left panel). Quantification of the soft agar colony formation assay results (right panel). **e** Knockout of TRIM38 significantly promoted glucose uptake, which could be restored by GLUT1 inhibition. **f** T24 cells (Ctrl & TRIM38-KO#1) with a Seahorse XF24 analyser for 100 min were used to depict the ECAR profiles. The metabolic inhibitors were injected sequentially at different time points as indicated. **g** EJ cells (Ctrl & TRIM38) with a Seahorse XF24 analyser for 100 min were utilized to show the ECAR profiles. The metabolic inhibitors were injected sequentially at different time points as indicated

To further figure out the associations between TRIM38 and GLUT1, we performed the lenti-virus infection technology to inhibit expression levels of GLUT1 in TRIM38-deficient cells. The CCK-8 assays indicated that knockdown of GLUT1 significantly suppressed cell growth in TRIM38-KO cells (T24, EJ and 5637) (Fig. 5A–C). Besides, the T24 cell soft agar colony formation efficiency was also markedly impaired when GLUT1 was inhibited, indicating that targeting GLUT1 could attenuated the colony formation ability of TRIM38-deficient cells (Fig. 5D). As a result, we investigated that whether TRIM38 deficiency could regulate glycolysis that promotes BLCA malignant processes. As shown in Fig. 5E, we found that knockout of TRIM38 could significantly enhanced glucose uptake and lactate production (Fig. 5E). Besides, GLUT1 inhibition could notably restore glycolytic activity and significantly decrease lactate production in TRIM38 knockout EJ cells (Fig. 5E). Subsequently, The extracellular acidification rate (ECAR) kinetic profiles further confirmed the remarkable increase of glycolytic activity in TRIM38-knockout T24 cells and the decrease in TRIM38-overexpressing EJ cells (Fig. 5F, G). Collectively, our results indicated that TRIM38/GLUT1 axis promotes BLCA tumourigenesis partly through the glycolytic pathway.

### Targeting GLUT1 (BAY-876) was effective to suppress progression of TRIM38^low^ bladder cancer

**Fig. 6:**
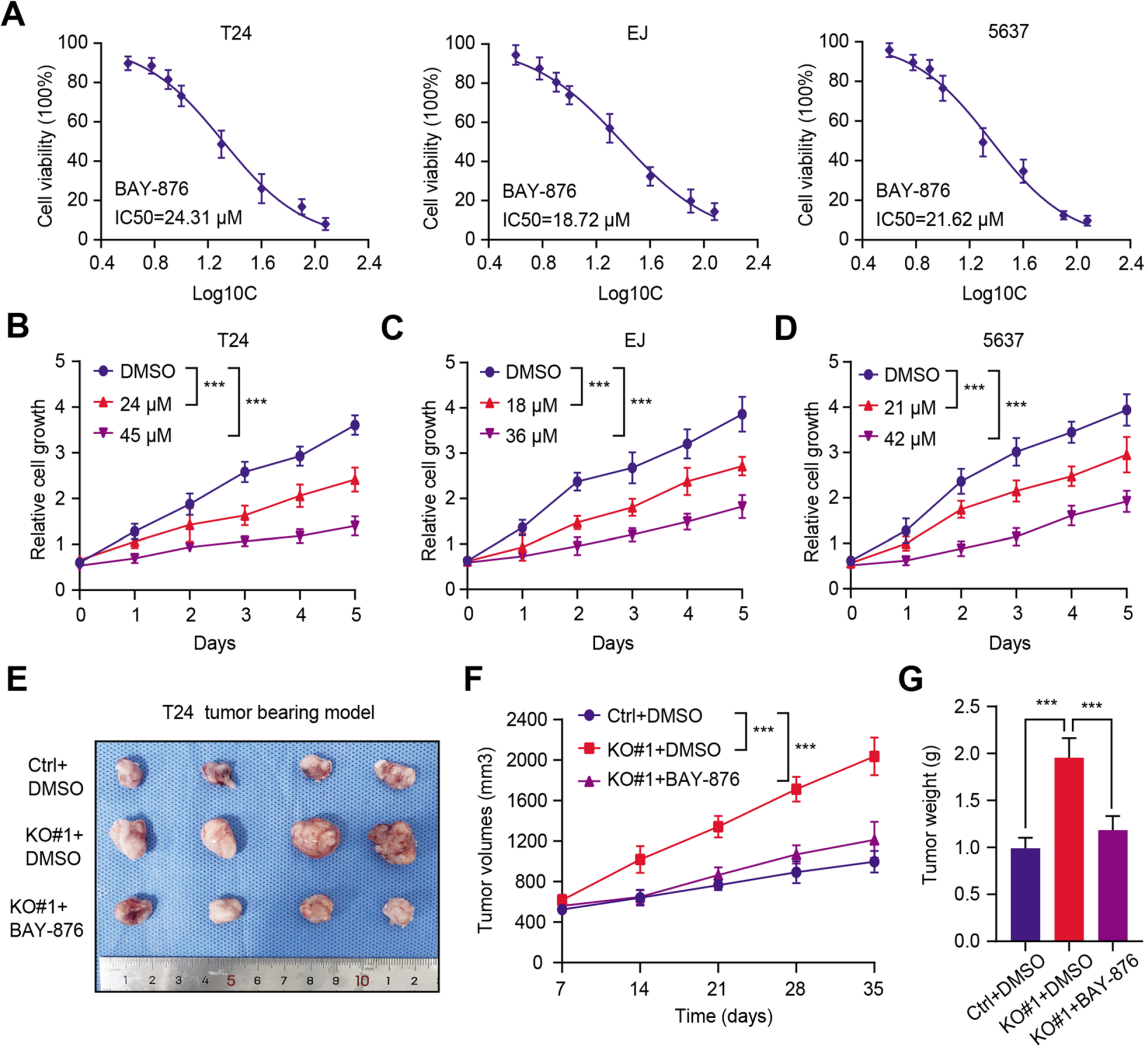
GLUT1 inhibitor (BAY-876) was effective to inhibit the progression of TRIM38^low/−^ bladder cancer. **a** The half maximal inhibitory concentration (IC50) values of BAY-876 (GLUT1 inhibitor) in BLCA cell lines (T24, EJ and 5637). **b**–**d** BAY-876 could suppress the cell growth of BLCA cells in a dose-dependent manner. **e** Representative image inhibited that BAY-876 could inhibit the tumor volumes derived from TRIM38-KO cells relative to tumors derived from control cells. **f** The tumor volumes were detected and recorded at the indicated timepoints and the growth curve was generated. **g** Tumors were resected and weighed to compare the differences in the indicated groups

Given that we have already found that low TRIM38 depended on accumulated GLUT1 to drive tumor progression, we thus considered whether GLUT1 inhibitor could be effective to suppress tumour growth in TRIM38^low^ BLCA. First of all, we confirmed and evaluated the half maximal inhibitory concentration (IC50) values of BAY-876 (GLUT1 inhibitor) in BLCA cell lines (T24, EJ and 5637) (Fig. 6A). Besides, we observed that BAY-876 could suppress tumor growth in a dose-dependent manner (Fig. 6B–D). Lastly, the tumor xenografts models were further established and BAY-876 was also effective to inhibit the growth of tumors derived from TRIM38-deficient cells, as indicated by the tumor volumes and tumor weight (Fig. 6E–G). Taken together, our findings suggested that targeting GLUT1 (BAY-876) is demonstrated to be an useful strategy to suppress BLCA tumor growth, especially for the TRIM38^low^ tumors.

## Discussion

Activation or suppression of the ubiquitin–proteasome system (UPS) has been shown to be associated with malignant features and drug sensitivity in various tumors [32, 33]. Besides, GLUT1-driven glycolytic reprogramming was demonstrated to be essential for tumor cell growth [34]. Given the clinical and functional importance of GLUT1 and its potential targeting vulnerability via drug inhibition in cancer treatment, it is of great importance to find the E3 Ub ligase that may target GLUT1 for ubiquitination. Here, in the current study, we conducted the UPS screening to thoroughly identify the prognostic UPS-signature that might be associated with the survival outcomes of BLCA. We validated that TRIM38 inhibition could enhance cell growth of BLCA and patients with low TRIM38 had a lower overall survival (OS) time relative to those with high TRIM38. Functional assays further confirmed that TRIM38 could restrict tumor growth and TRIM38 inhibition resulted in enhanced cell proliferation ability, migration efficiency and self-renewal potentiality. Mechanistically, we utilized the TAP/MS technology to identify that GLUT1 is the *bona fide* substrate of TRIM38. TRIM38 could interact with GLUT1 and promote GLUT1 degradation and ubiquitination. As a result, TRIM38 deficiency in BLCA leaded to abundance of GLUT1 proteins and depended on accumulated GLUT1 to drive BLCA progression, migration and enhanced glycolytic activity. Lastly, we found that GULT1 inhibitor (BAY-876) was effective to suppress BLCA growth and proliferation in vitro and in vivo, which could be a therapeutic vulnerability in TRIM38^low/−^ BLCA.

Intensive studies have previously demonstrated that high GLUT1 expression correlated with the extent of aggressiveness and vascular invasion in various tumors and was a predictive biomarker for disease recurrence [35, 36]. Chen et al. [37] found that METTL3 was the top essential m^6^A regulatory enzyme to induce GLUT1 translation in an m^6^A-dependent manner, which subsequently enhancing glucose uptake and the activation of mTORC1 signaling in colorectal cancer development. Besides, Wang et al. [38] observed that androgen receptor (AR) could directly bind to the GLUT1 gene promoter to promote GLUT1 transcription, which leading to castration resistance and enzalutamide resistance in prostate cancers. In addition, hypoxia could also enhance GLUT1 translocation to the plasma membrane, contributing to the glucose uptake in HUVEC [39]. However, we did not observe the differences of GLUT1 mRNA levels between BLCA and normal samples. However, elevated immunohistochemical staining intensities of GLUT1 was found in BLCA samples. We thus speculated that disorders of posttranslational modification may contribute to aberrant accumulation of GLUT1 proteins. However, little E3 ubiquitin ligases or deubiquitinating enzymes were previously reported to mediate the balance of GLUT1 proteins degradation. Based on the proteomics analysis and validations, we for the first time found the E3 ubiquitin ligase TRIM38 manipulated the ubiquitination and degradation of GLUT1, which could be a therapeutic target for following investigations. Supporting this, our results confirmed that GLUT1 inhibition (BAY-876) triggered the growth suppression of BLCA cells, especially for TRIM38^low^ cell lines. Meanwhile, we may speculate that TRIM38 expression levels may determine the cell sensitivity to BAY-876, which should be further investigated in a panel of BCLA cell lines. Though BAY-876 is proved to specifically target GLUT1, whether other small molecule GLUT1 inhibitors, like resveratrol, WZB11726, as well as salicylketoximes, have the similar or superior efficacy is promising to figure out. Lastly, we should further assess the in vivo safety of BAY-876 and the optimal doses for guiding clinical utility in the future.

Different from other family members, TRIM38 was less explored and reported to be involved in tumorigenesis. TRIM38 could function as an E3 ubiquitin or SUMO ligase, thereby targeting essential cellular signaling substrates, or as an enzymatic activity-independent regulator. TRIM38 expressed lowly in BLCA which was demonstrated to be a predictive bio-marker associated with prognosis. However, we were still unclear about the underlying mechanisms that explain down-regulated TRIM38 levels in BLCA. Besides, TRIM38 mediated the GLUT1 ubiquitination to influence BLCA tumorigenesis. Whether there existed other downstream targets of TRIM38 in BLCA that account for tumor progression is interesting to be thoroughly figured out.

However, there were still several shortcomings that need to be further improved in the current study. First of all, we only elucidated the associations between TRIM38 and glycolytic programming in BLCA. The underlying relationships between TRIM38 and immune infiltrations in BLCA tumor microenvironment (TME) were still unclear. Besides, large BCLA samples from local hospitals were warranted to further determine the prognostic significance of TRIM38. We need a relatively accurate cutoff to stratify TRIM38^high^ and TRIM38^low^ BLCA patients. Lastly, we demonstrated that TRIM38 could trigger GLUT1 degradation. Whether there existed other E3 ubiquitin ligases that alter GLUT1 proteins is needed to be explored in BLCA.

## Conclusion

Taken together, our results revealed that TRIM38 is an E3 Ub ligase for GLUT1 and mediates GLUT1 ubiquitination and degradation. TRIM38 inhibition enhances BLCA malignant features, including cell growth, migration and glycolysis capacity. Targeting GLUT1 (BAY-876) is an effective choice to suppress TRIM38^low/−^ BLCA progression, which reveals great significance for clinical translation.

## Supplementary Information

**Additional file 1: Table S1.** Summary of a list of UPS-related genes.

**Additional file 2: Table S2.** Univariate Cox regression analysis identifying survival-related UPS genes.

**Additional file 3: Table S3.** Clinical information of BLCA patients in three BLCA datasets.

**Additional file 4: Figure S1.** Detection of GLUT1 expressions in the TCGA-BLCA cohort. **a** Differential analysis was conducted and we did not find the differences of GLUT1 mRNA levels between normal and tumor samples. **b** Correlation analysis was conducted to assess the relationships between TRIM38 and GLUT1 mRNA levels in the TCGA-BLCA cohort.

## Data Availability

The data used to support the findings of this study are available from the corresponding author upon request.
