# Supplementary material for: TRIM38 triggers the uniquitination and degradation of glucose transporter type 1 (GLUT1) to restrict tumor progression in bladder cancer
Source: J Transl Med. 2021 Dec 14;19:508. doi: 10.1186/s12967-021-03173-x (PMC8670142; doi:10.1186/s12967-021-03173-x)
Supplement: Supplementary file 3 — Additional file 3: Table S3. Clinical information of BLCA patients in three BLCA datasets. [file 12967_2021_3173_MOESM3_ESM.docx]

**Table S3.** The brief summay of clinical characteristics of three databases in this study.

| ****Clinical****  ****Characteristics**** | ****Included datasets**** | | |
| --- | --- | --- | --- |
|  | ****TCGA**** | **[GSE13507](https://www.ncbi.nlm.nih.gov/geo/query/acc.cgi?acc=GSE13507)** | **[GSE32548](https://www.ncbi.nlm.nih.gov/geo/query/acc.cgi?acc=GSE32548)** |
| ****Total**** | 408 | 165 | 131 |
| ****Age**** |  |  |  |
| <60 | 87 | 42 | 26 |
| ≥60 | 321 | 123 | 105 |
| ****Gender**** |  |  |  |
| Female | 107 | 30 | 31 |
| Male | 301 | 135 | 100 |
| ****Grade**** | 10 cases missing |  | G1+G2 deemed as low grade |
| Low grade | 20 | 105 | 56 |
| High grade | 378 | 60 | 75 |
| ****AJCC stage**** | 2 cases missing | Calculated by 7th AJCC |  |
| 0a | 0 | 23 | NA |
| I | 2 | 80 | NA |
| II | 130 | 26 | NA |
| III | 140 | 29 | NA |
| IV | 134 | 7 | NA |
| ****T**** | 34 cases missing |  |  |
| Tis | 0 | 23 | 40 |
| T1 | 3 | 81 | 53 |
| T2 | 119 | 31 | T2+T3+T4 = 38 |
| T3 | 194 | 19 |  |
| T4 | 58 | 11 |  |
| ****N**** | 42 cases missing |  |  |
| N0 | 237 | 149 | NA |
| N1 | 46 | 9 | NA |
| N2 | 75 | 6 | NA |
| N3 | 8 | 1 | NA |
| ****M**** | 201 cases missing |  |  |
| M0 | 196 | 158 | NA |
| M1 | 11 | 7 | NA |
