# Supplementary figures and images for: TRIM38 triggers the uniquitination and degradation of glucose transporter type 1 (GLUT1) to restrict tumor progression in bladder cancer
Source: J Transl Med. 2021 Dec 14;19:508. doi: 10.1186/s12967-021-03173-x (PMC8670142; doi:10.1186/s12967-021-03173-x)

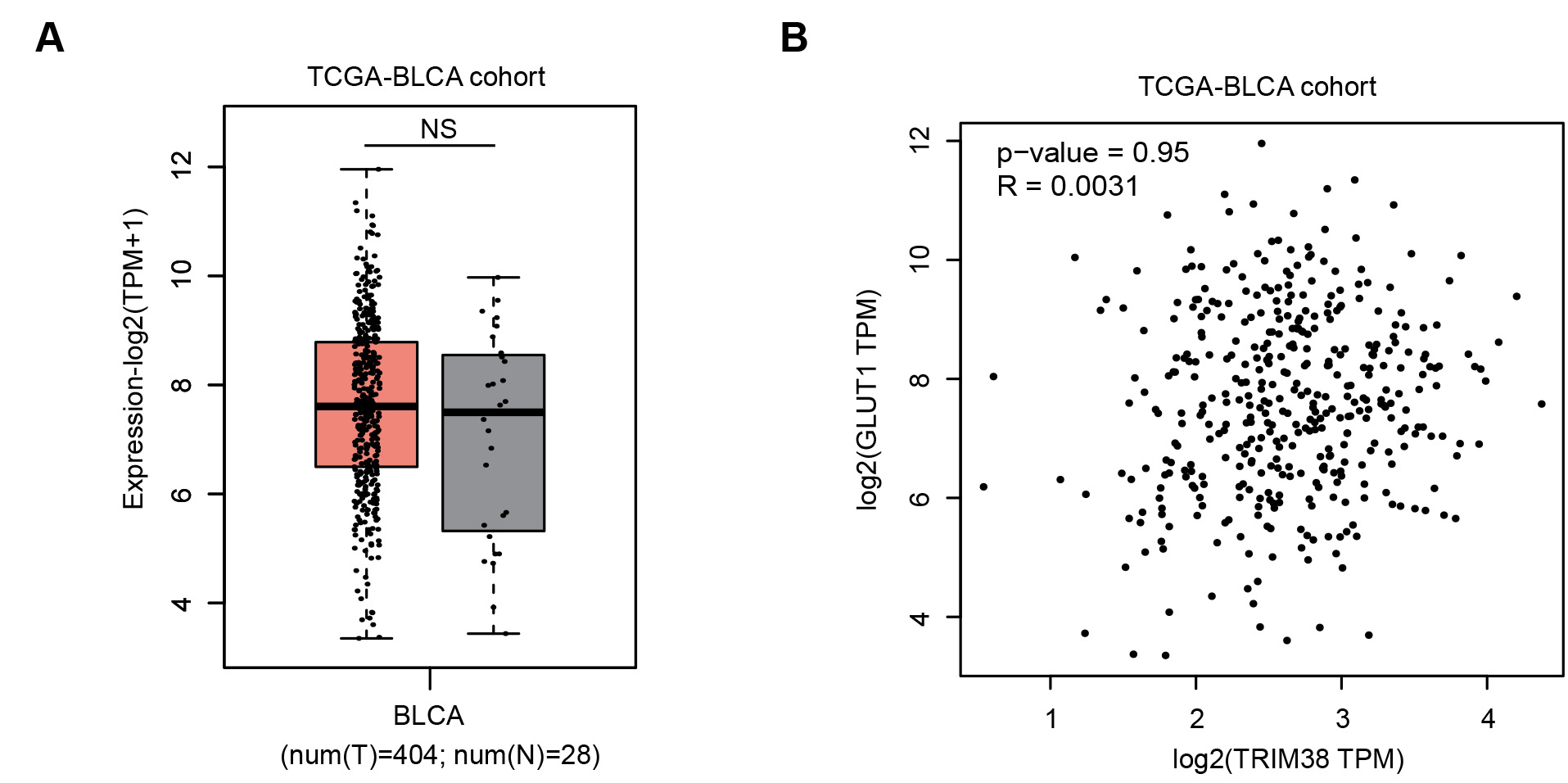

Supplement: Supplementary file 4 — Additional file 4: Figure S1. Detection of GLUT1 expressions in the TCGA-BLCA cohort. a Differential analysis was conducted and we did not find the differences of GLUT1 mRNA levels between normal and tumor samples. b Correlation analysis was conducted to assess the relationships between TRIM38 and GLUT1 mRNA levels in the TCGA-BLCA cohort. [file 12967_2021_3173_MOESM4_ESM.jpg]
